# Species of the genus *Mastrus* Förster (Hymenoptera, Ichneumonidae) of China with descriptions of two new species parasitizing sawflies (Hymenoptera)

**DOI:** 10.3897/zookeys.57.421

**Published:** 2010-09-21

**Authors:** Mao-Ling Sheng, Xiang-Fu Zeng

**Affiliations:** 1 General Station of Forest Pest Management, State Forestry Administration, 58 Huanghe North Street, Shenyang 110034, P. R. China; 2 Forest Pest Management and Quarantine Station of Hubei, Wuhan 430070, P. R. China

**Keywords:** Mastrus, new species, host, Argidae, Diprionidae, Tenthredinidae, Lepidoptera, Arge pullata, Diprion jingyuanensis, Pachynematus itoi

## Abstract

Four species of Mastrus Förster, 1869 are reported from China. Two, Mastrus nigrus Sheng & Zeng, **sp. n.** reared from Arge pullata (Zaddach) and Mastrus rugotergalis Sheng & Zeng, **sp. n.** reared from Diprion jingyuanensis Xiao & Zhang, are new to science. One, Mastrus deminuens (Hartig, 1838), is a parasitoid of Pachynematus itoi Okutani. A key to species of Mastrus Förster known in China is provided.

## Introduction

Mastrus Förster, 1869, belonging to the subfamily Cryptinae of Ichneumonidae (Hymenoptera), comprises 50 described species (Yu et al. 2005), of which 30 are known from the Palearctic, 16 from the Nearctic, one is Holarctic, one from the Neotropics, and two from the Oriental Region. The European species of the pictipes group of Mastrus were revised by Horstmann (1990).

The genus has not been studied thoroughly in China. Only two species, Mastrus deminuens (Hartig, 1838) and Mastrus ineditus (Kokujev, 1909), have been recorded (Kokujev 1909, Sheng and Chen 2001). In the present paper, two new species of Mastrus Förster, reared from Arge pullata (Zaddach) (Hymenoptera: Argidae) and Diprion jingyuanensis Xiao & Zhang (Hymenoptera: Diprionidae) collected from P. R. China, are reported.

## Methods

Materials used were collected using the following methods.

Rearing parasitoids. Cocoons of sawflies were collected from forests where there had been an outbreak of sawfly larvae lasting two or three years. Cocoons were stored individually in glass tubes with a piece of filter paper dipped in distilled water for maintaining moisture and plugged tightly with absorbent cotton at room temperature. Glass tubes are 60 mm long and 6 mm diameter. Emerged insects were collected daily.

Some European specimens of Mastrus deminuens (Hartig, 1838) for comparing with Chinese specimens mentioned in this article were provided by Dr. G. Broad and Dr. K. Horstmann. Photographs of the type of Mastrus ineditus were taken by Dr. A. Khalaim.

The morphological terminology mostly follows Townes (1969). Wing vein nomenclature is based upon Ross (1936) and the terminology of Mason (1986, 1990).

All specimens of Ichneumonidae and hosts (except those identified by Prof. Mei-Cai Wei preserved in Central South University of Forestry and Technology, P. R. China) are deposited in the Insect Museum, General Station of Forest Pest Management, State Forestry Administration, P. R. China.

## Descriptions

### Genus Mastrus

Förster, 1869

Mastrus Förster, 1869. Verhandlungen des Naturhistorischen Vereins der Preussischen Rheinlande und Westfalens, 25 (1868): 176. Type species: (Phygadeuon (Mastrus) neodiprioni Viereck) = aciculatus Provancher.

#### Diagnosis.

Fore wing vein 3rs-m absent. Hind wing vein 1-cu inclivous. Clypeus with pair of small teeth. Notaulus not reaching to center of mesoscutum. Propodeum completely areolated. Spiracle of first tergum a little behind middle. Upper valve of ovipositor with nodus, lower valve with distinct teeth.

#### Key to species of Mastrus known in China

**Table:** 

1.	First to fifth terga black. Antennae black or brownish black	2
–	Second and third terga brown to reddish brown. Antennae yellowish brown, at least basal half brown, apical half darker brown	3
2.	Malar space approximately as long as basal width of mandible. Postocellar line about 0.7 times as long as ocular-ocellar line. Mesopleuron with dense and strong transverse wrinkles. Tegulae, hind femora (except base) and tibiae black	Mastrus nigrus Sheng & Zeng, sp. n.
–	Malar space 1.2 times as long as basal width of mandible. Postocellar line about 1.1 times as long as ocular-ocellar line. Mesopleuron almost smooth medially. Tegulae yellowish to reddish. Hind femora yellowish brown distally, tibiae yellowish brown	Mastrus ineditus (Kokujev)
3.	Second tergum distinctly granulate and dull, or partly and obliquely granulate-strigose	Mastrus deminuens (Hartig)
–	Second tergum with more or less distinct longitudinal wrinkles	Mastrus rugotergalis Sheng & Zeng, sp. n.

#### Mastrus nigrus

Sheng & Zeng sp. n.

urn:lsid:zoobank.org:act:653E047B-0FF1-46B5-8F2F-5546BCF77B57

**Figures 1–6.**
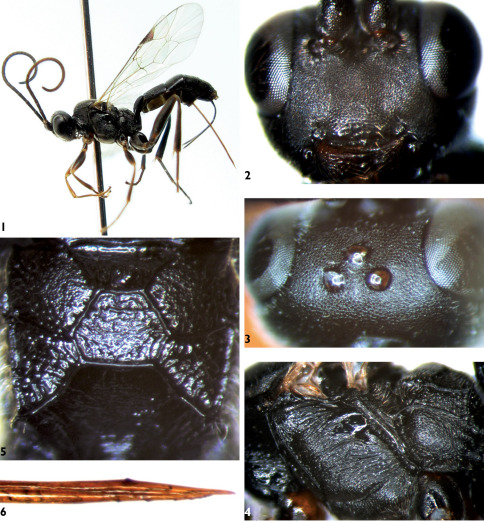
Mastrus nigrus Sheng & Zeng, sp. n. **1** Body, lateral view **2** Face **3** Vertex **4** Mesopleuron **5** Propodeum **6** Apical portion of ovipositor.

Figs 1, 2, 3, 4, 5, 6 

##### Etymology.

The specific name is derived from the body being entirely black.

##### Types.

*Holotype*, female, CHINA: Shennongjia, 2360m, Hubei Province, 15 April 2009, leg. MAN-QUN WANG. *Paratypes*: 2 females and 1 male, CHINA: Shennongjia, 2110 to 2360m, Hubei Province, 15 to 20 April 2009, MAN-QUN WANG.

##### Diagnosis.

Clypeal suture weak and indistinct; subapex of clypeus slightly raised. Hind wing vein 1-cu strongly inclivous, about 3.5 times as long as cu-a. Area superomedia of propodeum approximately as wide as long. Ventral profile of scape with distinct punctures. Tegulae black.

##### Description.

###### Female.

Body length 5.5–7.0 mm. Fore wing length 5.0–6.0 mm. Ovipositor sheath length 2.5–2.8 mm.

###### Head.

Face (Figure 2) 1.9–2.1 times as wide as long, with fine granulose texture and dense punctures, diameter of punctures 1.0–2.0 times distance between punctures, weakly convex centrally; upper margin medially concave. Clypeal suture weak and indistinct. Clypeus slightly convex, 2.5 times as wide as long; basal portion with texture as that of face, but medially sparsely punctate and apically smooth; subapex somewhat raised; apical margin with two small median protruberances. Mandible with dense punctures, upper tooth slightly longer than lower tooth. Median portion of cheek slightly rough, hind portion with punctures. Malar space approximately as long as basal width of mandible. Subocular sulcus indistinct. Gena with fine leathery texture and unclear punctures, about as long as middle width of compound eye. Vertex (Figure 3) with fine, unclear punctures, posterior portion with indistinct oblique lines. Postocellar line about 0.7 times as long as ocular-ocellar line. Upper portion of frons approximately flat, with texture as that of face; lower portion concave and shiny, with fine, transverse wrinkles. Antenna short, with 27 to 28 flagellomeres, apical portion not attenuated, ratio of length from first to fifth flagellomeres: 6.0:6.0:5.3:4.1:3.3. First and second flagellomeres about 3.75 times as long as widest diameter; ventral profile of scape with distinct punctures. Occipital carina complete.

###### Mesosoma.

Pronotum smooth, upper half of anterior margin with weak longitudinal wrinkles and punctures; median portion with dense oblique transverse wrinkles; upper posterior portion with weak, dense, oblique longitudinal wrinkles. Epomia weak. Mesoscutum evenly convex, with fine granulose texture, punctures indistinct. Notaulus evident on front portion of mesoscutum. Scutoscutellar groove smooth, with weak longitudinal wrinkles. Scutellum evenly convex, with fine granulose texture and fine punctures, diameter of puncture 1.0 to 2.0 times as long as distance between punctures. Postscutellum short, with fine punctures. Mesopleuron (Figure 4) with dense and strong transverse wrinkles, upper anterior corner with weak and short oblique wrinkles. Epicnemium with fine punctures, diameter of puncture approximately as long as distance between punctures. Epicnemial carina strong, upper end reaching to subalar prominence. Speculum small, with weak and fine transverse lines. Sternaulus sharp, reaching to hind margin of mesopleuron. Metapleuron rough, anterior portion with unclear transverse wrinkles, hind portion with unclear longitudinal wrinkles. Anterior section of juxtacoxal carina distinct, hind section unclear. Submetapleural carina complete and strong. Wings hyaline. Fore wing with vein 1cu-a distal of 1/M by about 0.3 times length of 1 cu-a. Vein 3rs-m absent; 2rs-m about 1.1 to 1.3 times as long as distance between it 2rs-m and 2m-cu. Vein 2-Cu approximately 2.0 times as long as 2cu-a. Hind wing vein 1-cu strongly inclivous, about 3.5 times as long as cu-a. Legs robust. Ratios of length of hind tarsomeres 1:2:3:4:5 are 10.0:4.0:3.0:1.7:2.0. Propodeum (Figure 5) completely areolate, carinae strong. Area basalis an inverse trapezium, with weak longitudinal wrinkles. Area superomedia hexagonal, approximately as wide as long, with very weak and fine transverse wrinkles. Costula connecting area superomedia distinctly behind its middle. Area petiolaris strongly sloping, with dense, oblique, transverse wrinkles. Area externa slightly coriaceous. Area dentipara, areae spiracularis and lateralis with irregular fine wrinkles. Area posteroexterna with strong longitudinal wrinkles. Spiracle circular, very small. Propodeal apophysis short and compressed.

###### Metasoma.

First tergum 1.8 times as long as apical width, with fine granulose texture, posterior portion with indistinct, fine, longitudinal wrinkles. Median dorsal carinae indistinct. Dorsolateral and ventrolateral carinae complete. Spiracle circular, very small, located slightly behind middle of first tergum. Second tergum approximately 0.7 times as long as apical width, with texture as that of mesoscutum. Third tergum 0.6 times as long as basal width, with fine granulose texture. Fourth and following terga strongly convergent backward. Ovipositor sheath 1.1 to 1.2 times as long as hind tibia. Ovipositor slightly compressed. Apical portion of lower valve with 7 weak ridges, basal 3 widely spaced, distal 4 moderately close together.

###### Color

(Figure 1). Black, except the following. Apical margin of pedicel yellowish brown. Ventral profile of apical portion of flagellomeres dark brown. Upper margin of mandible slightly reddish brown. Maxillary and labial palpi brownish black. Ends of front and middle trochanters, basal ends of all femora, apical portions of front and middle femora, front tibiae (at least ventral profiles), brown; middle tibiae blackish brown. Tarsi more or less brown. Posterior portion of sixth and seventh terga white. Stigma brownish black except white base. Veins blackish brown.

###### Male.

Body length about 6.0 mm. Fore wing length about 4.8 mm. Antenna with 23 flagellomeres, tenth and eleventh flagellomeres with linear tyloids. Ventral profile of scape, mandibles except teeth, maxillary and labial palpi, upper posterior corner of pronotum, tegulae, coxae and trochanters of front and middle legs buff. Front and middle legs sandy beige, except apices of tarsi slightly darkened. Hind femora and basal 0.7 of tibiae reddish brown, apical portion of each tarsal segment slightly brown.

##### Host.

Arge pullata (Zaddach) (Hymenoptera: Argidae).

##### Host plant.

Betula albo-sinensis Burkill (Betulaceae).

##### Biology.

The mature larva forms a cocoon outside the body of the larval host and inside the cocoon of the host.

##### Remarks.

This new species is similar to Mastrus ineditus but can be distinguished from the latter by the following combination of characters: clypeal suture weak and indistinct; subapex of clypeus slightly raised; ventral profile of scape with distinct punctures; costula connecting area superomedia distinctly behind its middle; tegulae black; middle and hind femora, tibiae and tarsi black or mostly black. Mastrus ineditus: clypeal suture distinct and deep; apical portion of clypeus depressed and lamelliform; ventral profile of scape almost smooth, punctures indistinct; costula connecting area superomedia at its middle; tegulae, apical portion of femora, tibiae and tarsi red.

#### Mastrus ineditus

(Kokujev, 1909)

Figs 7, 8, 9 

##### Notes.

No specimens were examined. Figures of the type, including the body (lateral profile), face, mesopleuron, wings, propodeum and ovipositor, were checked. Drafted figures by Dr. K. Horstmann were consulted.

**Figures 7–9.**
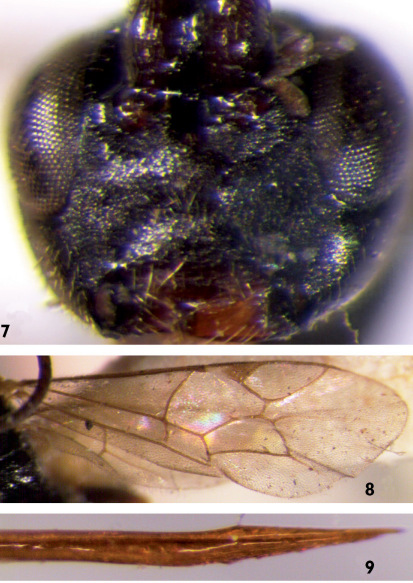
Mastrus ineditus (Kokujev, 1909). **7** Face **8** Wings **9** Apical portion of ovipositor.

##### Host.

Unknown.

##### Distribution:

China (Qinghai, Xizang) (Kokujev 1909, Chao 1976).

#### Mastrus deminuens

(Hartig, 1838)

##### Specimens examined.

1 female, CHINA: Dongfeng, Jilin Province, 15 May 2004, Mao-Ling Sheng.

##### Host.

Pachynematus itoi Okutani (Hymenoptera: Tenthredinidae).

##### Host plant.

Larix gmelinii (Rupr.) Rupr. (Pinaceae).

##### Distribution.

China (Jilin), Russia, Europe (Yu et al. 2005).

#### Mastrus rugotergalis

Sheng & Zeng sp. n.

urn:lsid:zoobank.org:act:7CAE315C-933F-45B0-AB7B-E37E5605607C

Figs 10, 11, 12 

##### Etymology.

The specific name is derived from the longitudinal wrinkles on the second tergum.

##### Types.

*Holotype*, female, CHINA: Qinyuan, Shanxi Province, 6 July 1996, Mao-Ling Sheng. *Paratypes*: 2 females, CHINA: Qinyuan, Shanxi Province, 5 June 1995. 4 females 2 males, CHINA: Qinyuan, Shanxi Province, 6 July 1996, Mao-Ling Sheng; 1 female, CHINA: Qinyuan, Shanxi Province, 12 June 1999, Guo-Fa Chen. 1 female (reared from a pupa of moth), CHINA: Weichang, 1673 m, Chengde, Hebei Province, 16 June 2010, Tao Li.

**Figures 10–12.**
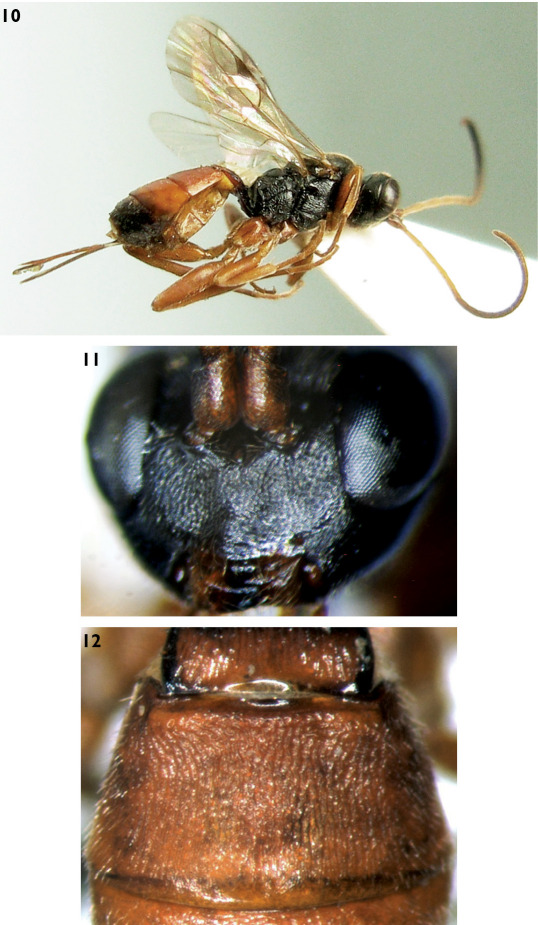
Mastrus rugotergalis Sheng & Zeng, sp. n. **10** Body, lateral view **11** Face **12** Second tergum.

##### Diagnosis.

Malar space 1.1–1.2 times as long as basal width of mandible. Hind wing vein 1-cu strongly inclivous, about 4.0 times as long as cu-a. Area superomedia 1.3 to 1.7 times as wide as long. Second tergum with longitudinal wrinkles. Tegulae darkish brown.

##### Description.

###### Female.

Body length 5.5–6.5 mm. Fore wing length 3.8–4.0 mm. Ovipositor sheath length 2.0–2.2 mm.

###### Head.

Face (Fig. 11) 1.9–2.0 times as wide as long, with granulose texture and indistinct fine punctures, diameter of punctures 0.5–2.0 times distance between punctures, evenly convex centrally; upper margin with a small median protuberance. Clypeal suture weak and unclear. Clypeus slightly convex, 2.2 times as wide as long; basal portion with indistinct fine punctures, apically smooth and sparsely punctate; apical margin with two small median protruberances. Mandible with indistinct sparse punctures, upper tooth distinctly longer than lower tooth. Cheek finely granulated. Malar space 1.1–1.2 times as long as basal width of mandible. Without subocular sulcus. Gena with fine leathery texture and fine punctures, about 0.9 times as long as middle width of compound eye. Vertex with texture as that of gena. Postocellar line about as long as ocular-ocellar line. Upper portion of frons approximately flat, with texture as that of face, but distinctly punctate; lower portion concave and shiny. Antenna with 23 to 24 flagellomeres, apical portion not attenuated, ratio of length from first to fifth flagellomeres: 5.2:5.0:4.2:3.3:2.9. First and second flagellomeres about 3.7 and 3.3 times as long as respective widest diameter. Occipital carina complete.

###### Mesosoma.

Anterior portion of pronotum smooth, upper portion with punctures; median portion with dense oblique transverse wrinkles; upper posterior portion with fine granulose texture, punctures distinct. Epomia weak. Mesoscutum with fine granulose texture, punctures indistinct. Notaulus evident on front portion of mesoscutum. Scutoscutellar groove nearly smooth, with weak longitudinal wrinkles. Scutellum shiny, evenly convex, with fine punctures, diameter of puncture 1.0 to 3.5 times as long as distance between punctures. Postscutellum transverse, anterolaterally deeply concave, punctures indistinct. Mesopleuron with dense and oblique transverse wrinkles, upper anterior corner with weak and short transverse wrinkles. Epicnemium with fine punctures, diameter of puncture approximately as long as distance between punctures. Epicnemium punctate indistinctly. Epicnemial carina strong, upper end reaching to subalar prominence. Speculum small, anterior portion with weak and fine transverse lines. Sternaulus sharp, reaching to hind margin of mesopleuron. Metapleuron slightly rough, with unclear oblique transverse wrinkles. Juxtacoxal carina anteriorly distinct, hind section unclear. Juxtacoxal and submetapleural carinae complete and strong. Wings brownish hyaline. Fore wing with vein 1cu-a distal of 1/M by about 0.3 times length of 1 cu-a. Vein 3rs-m absent; 2rs-m about 1.1 to 1.3 times as long as distance between it 2rs-m and 2m-cu. Vein 2-Cu approximately 2.0 times as long as 2cu-a. Hind wing vein 1-cu strongly inclivous, about 4.0 times as long as cu-a. Legs robust. Ratios of length of hind tarsomeres 1:2:3:4:5 are 10.0:4.2:3.0:1.7:3.0. Propodeum completely areolate, carinae strong. Area basalis inverse trapezium, almost smooth. Area superomedia hexagonal, 1.3 to 1.7 times as wide as long, slightly coriaceous. Costula connecting area superomedia at its apical 0.3. Area petiolaris strongly sloping, centrally rough, with short oblique wrinkles. Area dentipara with irregular fine wrinkles. Area posteroexterna with strong irregular wrinkles. Areae spiracularis and lateralis with irregular transverse wrinkles. Spiracle circular, very small, close to lateral longitudinal carina. Propodeal apophysis short and compressed.

###### Metasoma.

First tergum 1.4 to 1.6 times as long as apical width. Petiole with granulose texture. Postpetiole with fine, longitudinal wrinkles. Without median dorsal carinae. Dorsolateral and ventrolateral carinae complete. Spiracle circular, very small, slightly convex, located at apical 0.4 of first tergum. Second tergum (Fig. 12) approximately 0.6 times as long as apical width, with more or less distinct longitudinal wrinkles. Third tergum 0.6 times as long as basal width, 0.7 times as long as apical width, almost smooth. Fourth and following terga strongly convergent backward. Ovipositor sheath 1.1 to 1.2 times as long as hind tibia. Ovipositor not compressed, with weak nodus. Apical portion of lower valve with weak oblique ridges, basal 2 widely spaced.

##### Color

(Fig. 10). Black, except the following. Ventral profiles of scape and pedicel, flagellomeres 1 to 4 (5), legs except hind tibiae and tarsi slightly darkish brown, brown. Mandible yellowish brown, except teeth black. Maxillary and labial palpi dust-coloured. Longitudinal fleck of median portion of petiole, postpetiole, second and third terga, basal median portion of fourth tergum red. Tegulae darkish brown. Posterior margins of sixth and seventh terga and most of eighth tergum white. Stigma, except white base and veins, blackish brown.

Male. Body length about 5.0 mm. Fore wing length about 4.0 mm. Face with distinct punctures. Antenna with 21 flagellomeres, tenth and eleventh flagellomeres with linear tyloids. Ventral profiles of scape and pedicel whitish yellow. Tegulae buff. Middle and hind coxae blackish brown. Second and third terga brown with more or less irregular darkish fleck.

##### Host.

Diprion jingyuanensis Xiao & Zhang (Hymenoptera: Diprionidae). Pupa of moth (Lepidoptera).

##### Host plant.

Pinus tabulaeformis Carr. (Pinaceae).

##### Remarks.

This new species is similar to Mastrus deminuens but can be distinguished from the latter by the following combination of characters: face distinctly and densely punctate on a slightly granulate background; second tergum with more or less distinct longitudinal wrinkles, or finely granulose basally-centrally. Mastrus deminuens: face granulate and dull, with fine punctures; second tergum distinctly granulate and dull, or partly and obliquely granulate-strigose.

## Supplementary Material

XML Treatment for Mastrus
